# Oral Health Knowledge and Attitudes Towards Oral Health Education Among Elementary School Teachers in Kuwait

**DOI:** 10.3290/j.ohpd.b2259111

**Published:** 2021-11-05

**Authors:** Mishal A. Alshemari, Sarah A. Alkandari

**Affiliations:** a Dentist, Dental Division, Ministry of Health, Farwanyia Health District, Kuwait. Proposed and designed the study and collected the data. Interpreted the results, wrote the manuscript, critically revised it, read and approved the final manuscript.; b Dentist, Faculty of Dentistry, Health Sciences Centre, Kuwait University, Jabryia, Hawalli, Kuwait. Contributed to the acquisition of the data and performed the statistical analysis. Interpreted the results, wrote the manuscript, critically revised it, read and approved the final manuscript.

**Keywords:** attitude, elementary school teachers, knowledge, oral health education

## Abstract

**Purpose:**

While parents are the primary caregivers, school teachers can play a crucial role in shaping the personality and lifestyle of children during the elementary school years. The aims of this study were to evaluate the oral health knowledge of elementary school teachers and to assess their attitude towards oral health education and its implementation in the elementary school curriculum.

**Materials and Methods:**

A descriptive cross-sectional study was carried out among 1200 teachers working in government elementary schools. Data were collected through a structured, self-administered questionnaire consisting of teachers’ demographic data and their responses to knowledge and attitude questions.

**Results:**

A total of 1031 teachers participated in this study, with a response rate of 86%. About two-thirds (66%) of the participants demonstrated high oral health knowledge. Participants’ educational level was statistically significantly associated with their oral health knowledge. Almost all the participants (90%) agreed that oral health education should be included in the elementary school curriculum. More than two-thirds of the participants (74%) showed willingness to participate in online courses to promote oral health among elementary school children.

**Conclusion:**

Considering their oral health knowledge, positive attitude, teaching experience, and the fact that they have the potential to reach a large number of children and establish consistency and continuity in providing educational instructions, elementary school teachers should be encouraged to become involved in promoting oral health to elementary school children as a part of the teaching system. Such an approach is a good strategy to improve children’s oral health and therefore reduce the burden of preventable oral diseases – dental caries and periodontal disease – worldwide.

Even though oral diseases, particularly dental caries and periodontal disease, are largely preventable, they are still major public health problems throughout the world.^17^ About 60% to 90% of schoolchildren in most industrialised countries suffer from caries.^17^ According to the American Academy of Pediatrics, early childhood caries is about five times as common as asthma and seven times more common than hay fever.^7,9^ One study,^4^ conducted in 2006, demonstrated that 85% of six-year-old children in Kuwait were affected by caries. Furthermore, less than one-third of twelve- and fourteen-year-old Kuwaiti children had sound permanent dentition.^4^ A more recent study^5^ showed that the percentage of five- and six-year-old Kuwaiti children with caries-free primary dentition was 32% and 24%, respectively. Regarding periodontal disease, most schoolchildren in the world have signs of gingivitis.^17^ Furthermore, periodontal disease is the most common cause of tooth loss in adults.^7,9^ Honkala^12^ found that the oral hygiene habits of intermediate schoolchildren in Kuwait were far behind international recommendations.

At the beginning of the novel coronavirus disease 2019 (COVID-19) pandemic, dental clinics had to prioritise emergency and urgent care, delaying elective treatment to slow the spread of COVID-19 in dental-care settings as recommended by the Centers for Disease Control and Prevention (CDC).^10^ Providing preventive dental care during the COVID-19 pandemic, and in particular during the increased governmental restrictions including social distancing and nationwide quarantines, can be a challenging task. Moreover, many parents and caregivers have concerns about contracting COVID-19 and therefore postpone their children’s routine dental appointments.^8,11,16^

Another restriction measure the governments have undertaken in most countries to contain the spread of the novel coronavirus is to close schools and shift from the traditional in-person classes to online, virtual classes. Lessons learned at a younger age, especially those related to lifestyle and health choices, tend to stay with one through life. While parents are the primary caregivers, schoolteachers can play a crucial role in shaping the personality and lifestyle of children during the elementary school period.^13^ Moreover, schoolteachers can have provide a major contribution to promoting and implanting good oral health habits, as they can influence a large number of children.^13^ As demonstrated by several international studies,^1-3,14,15,18,19^ schoolteachers in different regions in the world had different levels of knowledge on oral health; however, the majority have still shown interest in providing oral health education to their students.

The current cross-sectional study aimed to evaluate the oral health knowledge of elementary school teachers in Kuwait. This study also assessed their attitude towards oral health and the implementation of oral health education in the elementary school curriculum.

## Material and Methods

### Ethical Considerations

This study was carried out in full accordance with the World Medical Association Declaration of Helsinki. It was approved by the ethics committee of the Ministry of Health as well as the Educational Research Administration at the Educational Research and Curricula Sector of the Ministry of Education in Kuwait. Permission to conduct the study was obtained from the head of the director manager’s office of each educational district as well as all the principals of the selected schools. Neither the name nor the contact information of the participating teachers was involved in the questionnaire; thus, participant confidentiality was maintained. After agreeing to participate, all subjects received verbal and written information about the nature and purposes of the study, and written informed consent was obtained.

### Study Design and Study Subjects

A descriptive cross-sectional study was carried out during the 2020/2021 academic year among elementary school teachers from six educational districts in Kuwait (all educational districts in Kuwait at the time of the study). Teachers working in government elementary schools were included in the current study. Those who were on leave or unwilling to participate were excluded from the study.

### Study Instrument

A structured, self-administered questionnaire was prepared by the authors. The questionnaire consisted of 26 closed-ended questions about the following variables: 1. demographic data such as age, gender, nationality, marital status, educational district, educational level, and years of teaching experience; 2. knowledge about caries and periodontal disease; 3. knowledge about oral hygiene practice and preventive dentistry, and 4. attitude towards oral health and oral health education. A pilot survey was carried out among 15 elementary school teachers to determine the validity of the questionnaire. The subjects completed the prepared questionnaire twice, with a one-week interval inbetween, to examine the reliability of the questions. Subjects in the pilot study were excluded from the main study sample.

### Sampling and Data Collection

Based on the total elementary school teacher population (24,123 teachers) in Kuwait at the time of the study and to have a confidence level of 95% and confidence interval of 3%, the sample size needed for this study was 1022. Considering a response rate of 85%, a total of 1200 teachers was targeted. The target sample was obtained using a stratified random sampling method. The six educational districts, i.e. the total number of educational districts in Kuwait at the time of the study, were used as the strata. Eight governmental elementary schools were then randomly selected from each educational district. The list of schools was obtained from the Ministry of Education. Electronic copies of the study questionnaire were sent through text messages to a random sample of 25 teachers in each selected elementary school to obtain the target sample (1200 teachers). Only completed surveys were eligible for submission and each participant was allowed to submit his/her response only once.

### Data Analysis

The data collected were analysed using the Statistical Package for Social Sciences software (SPSS v. 25; Chicago, IL, USA). Descriptive statistics were presented using frequency, percentage, mean ± SD, median, and range. Regarding the total knowledge score, each positive response was given a score of 1, and each negative response was given a score of 0. The individual scores were summed up to yield a total score with a maximum possible score of 12. After calculating the median, a total score less than the median was considered as low knowledge, and a total score equal to or higher than the median indicated high knowledge. To test for a statistically significant association between teachers’ knowledge and each of the different demographic variables, chi-squared tests, Pearson’s chi-squared test and Fisher’s Exact test, were used. A p-value < 0.05 was used as the cut-off level for statistical significance.

## Results

### Participants

**Fig 1:**
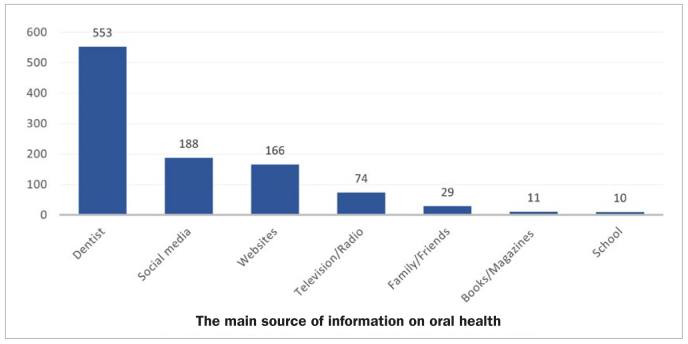
The main source of information on oral health upon which the participants’ rely (n = 1031).

A total of 1031 elementary school teachers participated in this study, giving a response rate of 85.9%. Table 1 summarises the demographic data of the study subjects. Participants’ mean age was 33.9 years (± 7.3 SD), ranging between 20 to 61 years. Most of the participants were females (91.8 %) and Kuwaitis (73.3%), whereas male teachers and non-Kuwaiti teachers comprised 8.2% and 26.7% of the study sample, respectively. Most elementary school teachers in this study had a graduate degree (91.7%). In addition, a majority of the participants had more than ten years (43.3%) of teaching experience, followed by zero to five years (34.1%), and a minority (22.6%) had six to ten years of teaching experience. Figure 1 shows the distribution of the participating teachers according to the main source of information on oral health. About half of the participants relied on the dentist for such information. Other sources were mainly social media and websites.

**Table 1:** Demographic characteristics of the participants (n = 1031)

Age	Mean ± SD (range)	
Age (years)	33.9 ± 7.3 (20 – 61)	
Demographic characteristic	Frequency	Percentage
**Gender**		
Male	85	8.2
Female	946	91.8
**Nationality**		
Kuwaiti	756	73.3
Non-Kuwaiti	275	26.7
**Marital status**		
Single	217	21.0
Married	753	73.0
Divorced	57	5.5
Widowed	4	0.4
**Educational district**		
Capital	178	17.3
Hawalli	191	18.5
Farwaniya	186	18.0
Jahra	150	14.5
Ahmadi	171	16.6
Mubarak Alkabeer	155	15.0
**Educational level**		
Graduate degree	945	91.7
Postgraduate degree	86	8.3
**Years of teaching experience**		
0-5 years	352	34.1
6-10 years >10 years	233	22.6
**Are you a parent?**	446	43.3
Yes	748	72.6
No	283	27.4

### Knowledge of Caries and Periodontal Disease

Table 2 shows the distribution of the participating teachers according to their knowledge of caries and periodontal disease. A majority (94.3%) of the elementary school teachers in this study knew that sugar is directly related to the onset of caries. While more than half (55.3%) of the participants defined dental plaque correctly, only 22.5% of them were aware that it is removed through toothbrushing and flossing. Moreover, most participating teachers (89.9%) identified bleeding gingiva as inflamed gingiva; however, only less than half of them (46%) answered its cause correctly. Almost one-third of the teachers (27%) stated that they did not know the cause of bleeding gingiva, and 21.1% thought that it is caused by frequent use of the toothbrush. The awareness that gingival bleeding can be prevented through regular toothbrushing and flossing was present in almost half of the participants (48.7%), while 20.3% thought it could be prevented by using mouthwash.

**Table 2:** Knowledge of dental caries and periodontal disease among the participants (n = 1031)

Variable	Frequency	Percentage
**Food directly related to the onset of caries**		
Fruits and vegetables	7	0.7
Sugars*	972	94.3
Carbohydrates	30	2.9
Fats	7	0.7
Do not know	15	1.5
**Dental plaque**		
Food residues on tooth surfaces	202	19.6
A sticky film of bacteria on tooth surfaces*	570	55.3
Staining on teeth	145	14.1
Do not know	114	11.1
**Removal of dental plaque**		
Through professional scaling	587	56.9
With the use of toothbrush and dental floss*	232	22.5
With the use of fluoride mouthwash	76	7.4
Do not know	136	13.2
**Bleeding gums means**		
Gums are healthy	5	0.5
Gums are inflamed*	927	89.9
Allergic reaction to certain food	61	5.9
Do not know	38	3.7
**Cause of bleeding gum**		
Hot drinks	33	3.2
Spicy food	27	2.6
Frequent use of toothbrush	218	21.1
Infrequent use of toothbrush (poor oral hygiene)*	474	46.0
Do not know	279	27.1
**Bleeding gums can be prevented**		
Through using vitamins	97	9.4
Through using mouthwash	209	20.3
Through brushing and flossing*	502	48.7
Through using the toothbrush as few times as possible	39	3.8
Do not know	184	17.8

*Correct answer.

### Knowledge of Oral Hygiene Practice and Preventive Dentistry

The distribution of the elementary school teachers in the current study according to their knowledge on oral hygiene practice and preventive dentistry is demonstrated in Table 3. Of the participating teachers, 35% and 53.7% stated that the recommended frequency of toothbrushing is twice daily and three times daily, respectively. Moreover, 35.6% of the teachers were aware that the recommended toothbrushing time is two minutes, while 33.9% of the teachers thought that one minute was the recommended time. Almost half of the participants (49.1%) knew that a soft toothbrush is the brush type recommended; however, the other approximate half (47%) thought that a medium toothbrush is the recommended type. In addition, almost half of the participants (49.4%) knew that the recommended frequency of toothbrush change is every three months or sooner if the toothbrush bristles become frayed with use. More than two-thirds of the teachers (71.2%) correctly identified the recommended amount of toothpaste to be applied on the toothbrush. Furthermore, more than half of the teachers (64.9%) were aware that the recommended frequency of regular dental visits is every six months, while 15.2% and 11.3% of the teachers thought that the child should be taken to the dentist every three months and whenever s/he complains of dental pain, respectively.

**Table 3:** Knowledge of oral hygiene practice and preventive dentistry among the participants (n = 1031)

Variable	Frequency	Percentage
**Recommended frequency of brushing**		
Once daily	5	0.5
Twice daily*	361	35.0
Three times daily	554	53.7
More than three times daily	62	6.0
Do not know	49	4.8
**Recommended brushing time**		
Less than one minute	72	7.0
One minute	349	33.9
Two minutes*	367	35.6
More than two minutes	143	13.9
Do not know	100	9.7
**Recommended type of toothbrush**		
Soft*	596	49.1
Medium	485	47.0
Hard	7	0.7
Do not know	33	3.2
**What is the recommended frequency of toothbrush change if the bristles become frayed with use?**		
Monthly	509	24.1
Every three months*	196	49.4
Every six months	5	19.0
Yearly	73	0.5
Do not know		7.1
**Recommended amount of toothpaste that should be applied on the toothbrush**		
Equivalent to pea size*	734	71.2
Covering the entire toothbrush	263	25.5
Amount that will produce abundant foam	5	0.5
Do not know	29	2.8
**The child is taken to the dentist**		
When the child complains of dental pain	116	11.3
Every 3 months	157	15.2
Every 6 months*	669	64.9
Once every year	43	4.2
Do not know	46	4.5

*Correct answer; based on the American Dental Association (ADA) and the American Association of Pediatric Dentistry (AAPD) guidelines.

### Total Knowledge and its Association with Participants’ Characteristics

**Figure 2:**
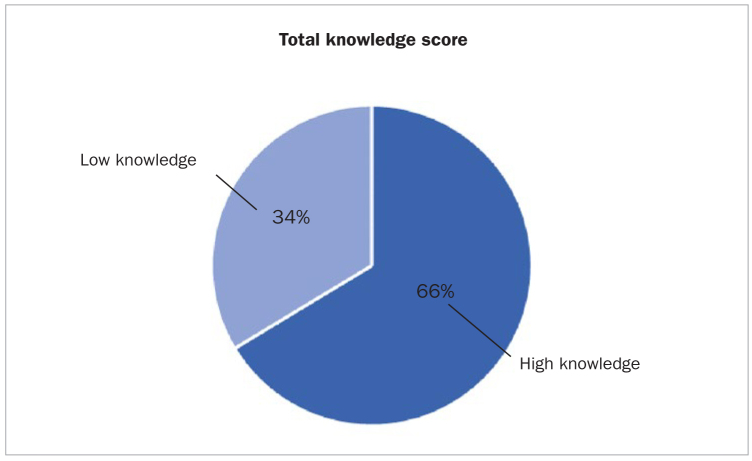
Total knowledge among participants (n = 1031).

The total oral health knowledge mean score for the participants was 6.2 (± 1.9 SD) with a median score of 6, ranging from 0 to 12 for 12 questions. Of the 1031 elementary school teachers in this study, 684 teachers (66%) demonstrated high knowledge (Fig 2). As shown in Table 4, female teachers had higher scores than male teachers; however, this finding is not statistically significant (p = 0.05). The educational level also had a statistically significant association, as teachers with a graduate degree (Master’s, PhD) showed higher total knowledge scores compared to those with an undergraduate degree (p = 0.035). In contrast, no statistically significant association was found regarding teachers’ age, nationality, marital status, years of teaching experience, and educational district.

**Table 4:** Association of the total knowledge with the different characteristics of the participants (chi-squared test)

Variable	Total knowledge		p-value
	High knowledge (n = 684)	Low knowledge (n = 347)	
	n (%)	n (%)	
**Age**			
20-29 years	224 (66.5)	113 (33.5)	0.61
30-39 years	291 (66.1)	149 (33.9)	
40-49 years	156 (67.8)	74 (32.2)	
≥50 years	13 (54.2)	11 (45.8)	
**Gender**			
Male	24 (28.2)	61 (71.8)	0.05
Female	660 (69.8)	286 (30.2)	
**Nationality**			
Kuwaiti	507 (67.1)	249 (32.9)	0.42
Non-Kuwaiti	177 (64.4)	98 (35.6)	
**Marital status**			
Single	136 (62.7)	81 (37.3)	0.07
Married	499 (66.3)	254 (33.7)	
Divorced	46 (80.7)	11 (19.3)	
Widowed	3 (75.0)	1 (25.0)	
**Educational district**			
Capital	129 (72.5)	49 (27.5)	0.07
Hawalli	129 (67.5)	62 (32.5)	
Farwaniya	130 (69.9)	56 (30.1)	
Jahra	86 (57.3)	64 (42.7)	
Ahmadi	112 (65.5)	59 (34.5)	
Mubarak Alkabeer	98 (63.2)	57 (36.8)	
**Educational level**			
Graduate degree	618 (65.4)	327 (34.6)	0.04*
Postgraduate degree	66 (76.7)	20 (23.3)	
**Years of teaching experience**			
0-5 years	227 (64.5)	125 (35.5)	0.66
6-10 years	157 (67.4)	76 (32.6)	
>10 years	300 (67.3)	146 (32.7)	
**Are you a parent?**			
Yes	496 (66.3)	252 (33.7)	1.00
No	188 (66.4)	95 (33.6)	

*Significant at p<0.05.

### Attitude Towards Oral Health and Oral Health Education

Table 5 summarises the responding teachers’ attitude towards oral health and oral health education. More than two-thirds of the teachers (72.6%) in the present study believed that oral health is related to general health. Most of the participating teachers (82.3%) agreed that it is necessary to treat caries in the primary dentition. Moreover, the majority of the teachers (90.0%) showed a positive attitude towards the implementation of oral health education in the elementary school curriculum. The majority (81.5%) also agreed that all elementary school teachers should have training in oral health education. In addition, more than two-thirds of the teachers (74.4%) were willing to participate in online/virtual classes to promote oral health among elementary school students.

**Table 5:** Attitude towards oral health and oral health education among the participants (n = 1031)

Variable	Frequency	Percentage
**Oral health is not related to general health**		
Agree	170	16.5
Uncertain	113	11.0
Disagree	748	72.6
**It is necessary to treat tooth decay in primary teeth (baby teeth)**		
Agree	849	82.3
Uncertain	135	13.1
Disagree	47	4.6
**Oral health education should be included in the elementary school curriculum**		
Agree	937	90.9
Uncertain	52	5.0
Disagree	42	4.1
**All elementary school teachers should have training in oral health education**		
Agree	840	81.5
Uncertain	90	8.7
Disagree	101	9.8
**Are you willing to assist the dentist and participate in online/virtual classes to promote oral health?**		
Yes	767	74.4
Uncertain	123	11.9
No	141	13.7

## Discussion

This study evaluated the oral health knowledge among teachers working in government elementary schools in Kuwait, as well as their attitude towards oral health and the implementation of oral health education in the elementary school curriculum. Most of the participating teachers were females, because at the time of the study, 92.7% (22,356 out of 24,123 teachers) of elementary school teachers in Kuwait were women. About two-thirds (66%) of the teachers in the present study demonstrated high knowledge of oral health. This finding was in accordance with other studies conducted in other regions,^1-3,14,15,18,19^ which concluded that elementary school teachers had acceptable oral health knowledge. According to the participating teachers, the main source of information on oral health was the dentist, followed by social media and websites. A study^6^ conducted among the Saudi population found that accessibility, ease of use, and being free of charge are the main reasons for using social media as a source of oral health information.

When the total oral health knowledge was correlated with educational level, participating teachers who had a higher educational level obtained higher scores. This finding emphasises the importance of providing elementary school teachers with educational programs and workshops on oral health.

With respect to teachers’ attitude, the ﬁndings of the present study were similar to those presented in the literature,^1,2,13,15,18,19^ showing an average above 70%. Most of the participants in this study had a positive attitude towards oral health. Also, the majority agreed that oral health education must be implemented in the elementary school curriculum, and elementary school teachers should have training in oral health education. In addition, more than two-thirds of the subjects showed a willingness to participate in virtual classes to promote oral health among elementary school students.

Considering their oral health knowledge, positive attitude, teaching experience, and the fact that they have the potential to reach a large number of children and establish consistency and continuity in providing educational instructions, elementary school teachers should be encouraged to become involved in promoting oral health to elementary school children. Such an approach is a good strategy to improve children’s oral health and therefore reduce the burden of the preventable oral diseases caries and periodontal disease in Kuwait.

The present study was carried out during the COVID-19 pandemic period when governmental restrictions and social distancing measures were undertaken. Thus, data were collected using a self-reported electronic survey. Therefore, response bias is a potential limitation of this study.

## Conclusion

This study demonstrated that two-thirds of elementary school teachers in Kuwait had an adequate level of knowledge on oral health. Almost all of them showed a positive attitude towards the implementation of oral health education in the elementary school curriculum and more than two-thirds of them were willing to participate in online classes to promote oral health among elementary school children. The present study also showed that educational level significantly influenced the oral health knowledge of the participants. Therefore, it is highly recommended to provide elementary school teachers with a guide manual that is well prepared by dental professionals in Kuwait. Also, the collaboration between the Dental Sector of the Ministry of Health and the elementary School Curricula Sector of the Ministry of Education is advised to include oral health education in the elementary school curriculum in Kuwait.
